# Prevalence and predictors of midwakh smoking among male students of Qassim University, Al-Qassim, Saudi Arabia

**DOI:** 10.18332/tid/125725

**Published:** 2020-09-04

**Authors:** Faisal Almogbel, Salman Almuqbil, Unaib Rabbani, Yasser Almogbel

**Affiliations:** 1 Department of Infection Control, King Fahad Specialist Hospital, Buraidah, Saudi Arabia; 2 Dental Department, Jazan Armed Forces Hospital, Jazan, Saudi Arabia; 3 Quality and Accreditation Unit, Family Medicine Academy, Buraidah, Saudi Arabia; 4 Department of Pharmacy Practice, College of Pharmacy, Qassim University, Buraidah, Saudi Arabia

**Keywords:** midwakh, smoking, Saudi Arabia, students

## Abstract

**INTRODUCTION:**

Novel tobacco products are becoming more popular in the Middle East and especially in Saudi Arabia. We studied the prevalence of midwakh pipe (tobacco smoking pipe) use among students at Qassim University and evaluated factors associated with midwakh pipe usage.

**METHODS:**

A cross-sectional study was conducted using a self-administered questionnaire to collect the data. The participants were male students from Qassim University, Al-Qassim, Saudi Arabia. Data were collected over the period from May to June 2018. A multivariable logistic regression model was used to identify sociodemographic factors and smoking history related to midwakh smoking.

**RESULTS:**

A total of 316 responses were collected; 7.9% (n=25) were midwakh smokers at some point, and 3.8% (n=12) were current midwakh smokers. Multivariable logistic regression indicated that the significant predictors of midwakh smoking among male university students were having a friend who is a tobacco or midwakh smoker, and consuming other forms of tobacco other than midwakh.

**CONCLUSIONS:**

Having midwakh smoking friends, having friends smoking any other form of tobacco, and being a smoker could predict the use of midwakh among university male students in Saudi Arabia. Interventions are required to control this behavior to prevent smoking-related health consequences.

## INTRODUCTION

Smoking is considered the leading cause of global preventable, premature death. As a result, tobacco consumption has been labeled one of the major threats to health. According to statistics in Saudi Arabia, around 28000 smokers died annually between 2001 and 2010 due to heavy smoking^1^. The total economic loss caused by tobacco in Saudi Arabia has been estimated to be between 22.6 and 25.6 billion US$ between 2000 and 2010^1^. Socially, smoking tobacco is considered unacceptable among men and more so among women^2,3^. The Islamic religion prohibits any action that might negatively affect health, such as smoking or taking drugs^2^.

Cigarette consumption continues to rise worldwide^4^, and tobacco consumption is increasing in Saudi Arabia^1,5^. In 2010, the World Health Organization (WHO) estimated that about 16% of Saudi Arabia’s population smoked (approximately 3.1 million people)^6,7^. The WHO Eastern Mediterranean Region (EMRO) has the highest growth rate in the cigarette market, even though significant reductions in smoking rates have occurred in the United Kingdom, Australia, Brazil, and other countries^4^. Several studies on tobacco smoking reported different prevalence percentages of smoking. This discrepancy may be related to several factors such as data collection methods, geographical locations, age ranges, gender, and education level^8-10^. In a recent meta-analysis evaluating 29 studies on the prevalence of tobacco smoking (including cigarettes, waterpipes, and cigars) among Saudi college students, it was found that 17% smoked^11^. It was also higher than that of neighboring countries such as United Arab Emirates (UAE), Yemen, and Iran, where 15.1%, 12.4%, and 11.6% of the student population were smokers, respectively^12-14^.

Men at college age are at risk of indulging in risky behaviors, predisposing them to injuries, diseases, and death^15,16^. The United States Substance Abuse and Mental Health Services Administration reported that the rate of tobacco consumption (including cigarettes, cigars, pipe tobacco, smokeless tobacco, chewing tobacco, and snuff) doubled among young adults between 18 and 25 years of age compared to those aged ≥26 years from 2002 to 2009^17^.

A midwakh is a small pipe composed of a mouthpiece stem and a bowl for smoking tobacco^18,19^. It originated in Northern Iran in the 15th century and gained popularity among sailors in the Middle East in the 1500s. The midwakh’s bowl is filled with dokha, which is a tobacco blend that contains a combination of tobacco leaves with a variety of spices, herbs, dried flowers, or dried fruit. The pipe can hold approximately 5 g of tobacco leaves^20^. In a study conducted on 159 adults, researchers found no significant difference between cigarettes and midwakhs with respect to levels of exhaled carbon monoxide or those of cotinine, a metabolite of nicotine in the saliva^20^. Typically, smokers require only two to three inhalations before the dokha needs to be refilled entirely^20,21^. Midwakh smoking is prevalent in the UAE and growing^22^. Midwakh smoking may emerge in Western countries among youth subcultures, as evidenced by the acceleration of websites selling midwakhs and dokha, and by anecdotal observations of midwakh and dokha sales in Western specialty stores^19^.

A study by Shaikh et al.^20^ was conducted to evaluate the acute effects of dokha smoking in a group of healthy university students. A sample of 97 men aged ≥17 years were included in the study. They observed an increase in systolic blood pressures (12 ± 1 mmHg), heart rates (20 ± 2 bpm), and respiratory rates (4 ± 1 breaths/min)^21^. Al-Houqani et al.^22^ analyzed 170430 UAE nationals aged ≥18 years. The prevalence of smoking was found to be 24.3% in men and 0.8% in women. Cigarette smoking was the most common at 77.4% followed by midwakhs at 15%^22^. Jayakumary et al.^23^ focused on the prevalence and patterns of dokha smoking among medical and allied health students in Ajman, UAE. A total of 104 students were included in this study. The prevalence of dokha smoking was found to be 30.4% in men and 5.1% in women. Crookes and Wolff^24^ focused on 394 high school students from English curriculum schools in Dubai, UAE. About 30% of the boys were tobacco users compared to 15.4% of girls. About 54.8% of smokers were consuming dokha^24^. Another study conducted among 560 secondary school students in Ajman, UAE, found that the percentage of participants who ever smoked tobacco was 39%, with 36% for smoking dokha. The percentage of students who currently smoked dokha was 24%^25^. Asfour et al.^26^ examined risky behaviors among 439 expatriate teenagers living in the UAE: 20% of male teenagers had smoked dokha at least once, and 7% of female teenagers reported that they had tried dokha^26^. Aden et al.^27^ examined the prevalence of cigarette, waterpipe, and midwakh usage among applicants to Abu Dhabi’s Premarital Screening program in 2011. The percentage of those who currently smoked was 24.7%. Most of the participants had smoked cigarettes (11.5%), midwakhs (5.9%), and waterpipes (4.8%), and 2.5% had smoked a combination^27^. Siddiqua et al.^28^ conducted a cross-sectional study from 2007 to 2009 on 505 adolescents, including nationals and expatriates living in the United Arab Emirates and aged 13–20 years. The prevalence of smoking midwakhs was 12.1% in men and 1% in women. Factors associated with midwakh smoking included gender, tobacco smoking, and illegal drug use or experimentation^28^. Another study was conducted in Leba among 7th to 12th grade students and found that about 5% of the students were midwakh smokers, most of them males^29^.

Whereas knowledge regarding predictors of tobacco use is abundant, little is known about the factors predicting midwakh use. Although several papers have addressed midwakh smoking in the United Arab Emirates, no data have been published for Saudi Arabia, to the best of our knowledge.

The main goal of this study was to assess the factors that predict midwakh usage among male college students in Saudi Arabia, bridging this gap in the literature. Gaining knowledge about the predictors of midwakh use among college students is essential for developing effective prevention measures. These results add to the literature and will help university administrators and public health specialists develop effective prevention programs.

## METHODS

### Study design and procedure

This cross-sectional study investigated the prevalence and predictors of midwakh smoking among the male student population of Qassim University (Al-Qassim, Saudi Arabia). A convenience sampling method was used to obtain responses from students. Female students were not included because smoking is not socially acceptable in Saudi Arabia^2,3^. Ethical approval was obtained from the regional ethics committee in the Al-Qassim region. Faculties in different colleges were approached, and institutional review board approval obtained to participate in this study. All the instructors who agreed to participate in the study were asked to allocate 20 minutes during class time for the survey to be completed. All the surveys were collected in a sealed box. Informed consent was obtained from all subjects prior to their participation. No identifying information was collected. The minimum required sample size for two-tail logistic regression was 305, based on the following assumptions: 1) α-level = 0.05, 2) power = 80%, and 3) odds ratio or effect size = 1.4. An electronic, self-administrated questionnaire was used for data collection. The survey was adopted and back-translated from a pretested validated survey^9,10^. To ensure face validity, the translation was performed by two independent translators who were specialists in public health. The questionnaire was pilot-tested among 20 participants to ensure its clarity. The questionnaire included 33 questions divided into three domains: demographic and socioeconomic characteristics, tobacco use history, and factors associated with midwakh use. The demographic and socioeconomic characteristics included age, marital status, monthly income, and academic performance. Tobacco use history assessed the frequency of substances used, both within one’s lifetime and in the last 30 days. Questions on tobacco use asked about the following substances: cigarettes, electronic cigarettes, waterpipes, electronic waterpipes, and cigars. Factors associated with midwakh use evaluated the effects important people had on the respondent’s life, such as immediate family members or close friends whose smoking behaviors might influence the participant’s smoking behavior. Tobacco smoking was defined as inhalation and exhalation of burned tobacco fumes containing nicotine^17^. Current smokers were defined as students who had used tobacco products on at least one day within the past month. Students were asked if they had tried at least one puff of tobacco with a midwakh in the past 30 days^30^ . Current smokers were defined as students who smoked at least one cigarette per day over the past 30 days^13,14^.

Analyses were performed using the Statistical Package for the Social Sciences (IBM SPSS version 22, IBM SPSS Inc., OK, USA). Chi-squared and Fisher’s exact tests were used to calculate associations. Variables (p≤0.05) in bivariate analyses were included in the logistical order used to arrive at the final models^31^. A multivariate logistical regression model was fitted to identify factors associated with midwakh usage. All tests were two-sided, and a p<0.05 was considered significant.

## RESULTS

A total of 316 respondents were included in the study. Of these, 25 participants (7.9%) reported that they had smoked a midwakh at least once. Of the students who had tried midwakh at least once, twelve students (48%) reported that they were current midwakh smokers. The mean age of the respondents was 21.2 ± 2 years with a median of 20.5 years; 204 (64.5%) were more than 21 years old. Approximately 1% of respondents were married and 40% had excellent academic performance. The results are shown in Table 1.

**Table 1:** Comparison of sociodemographic characteristics and smoking status between those who had ever smoked midwakh and non-midwakh smokers (N=316)

*Characteristics*	*Total*		*Non-smokers (n=291)*	*Smokers (n=25)*	*p*
	*n*	*%*	*n (%)*	*n (%)*	
**Age** (years)					
>21	112	35.5	99 (88.4)	13 (11.6)	0.071
≤21	204	64.5	192 (94.1)	12 (5.9)	
**Marital status**					
Not married	313	99.1	289 (92.3)	24 (7.7)	0.220*
Married	3	0.9	2 (66.7)	1 (33.3)	
**Academic performance**					
Excellent	126	39.9	116 (92.1)	10 (7.9)	0.425*
Very good	118	37.3	110 (93.2)	8 (6.8)	
Good	58	18.4	54 (93.1)	4 (6.9)	
Acceptable	14	4.4	11 (78.6)	3 (21.4)	
**Academic level**					
High	245	77.5	227 (92.7)	18 (7.3)	0.490
Average and low	71	22.5	64 (90.1)	7 (9.9)	
**Monthly income (SAR)**					
<2000	251	79.4	229 (91.2)	22 (8.8)	0.438 *
≥2000	65	20.6	62 (95.4)	3 (4.6)	
**Have a smoking father**					
No	309	97.8	287 (92.9)	22 (7.1)	0.012 *
Yes	7	2.2	4 (57.1)	3 (42.9)	
**Have a smoking mother**					
No	302	95.6	281 (93)	21 (7)	0.004 *
Yes	14	4.4	10 (71.4)	4 (28.6)	
**Have a smoking sibling**					
No	204	64.6	191 (93.6)	13 (6.4)	<0.001
Yes	112	35.4	100 (89.3)	12 (10.7)	
**Have a smoking friend**					
No	285	90.2	277 (97.2)	8 (2.8)	<0.001
Yes	31	9.8	14 (45.2)	17 (54.8)	
**Have a midwakh smoking friend**					
No	157	49.7	155 (98.7)	2 (1.3)	<0.001*
Yes	159	50.3	136 (85.8)	23 (14.5)	
**Consume other forms of tobacco**					
No	212	67.1	209 (98.6)	3 (1.4)	<0.001*
Yes	104	32.9	82 (78.8)	22 (21.2)	

* Fisher’s test p≤0.05. SAR: 1000 Saudi Arabia Riyal about 270 US$.

A chi-squared analysis was conducted, as shown in Table 1. Approximately 11.6% of the participants who had ever smoked a midwakh (ESM) were more than 21 years old. There was no significant association between age and ever having smoked a midwakh [χ^2^(1)=3.25; n=316; p=0.071]. In terms of marriage, 7.7% of the participants who reported ever smoking a midwakh were unmarried. About 4.6% of participants with a monthly income of ≥2000 SAR (1000 Saudi

Arabia Riyal about 270 US$) reported that they had ever smoked a midwakh. Of those reporting ‘excellent’ academic performance, 7.7% were smokers. There was no significant association between academic performance and ever having smoked a midwakh (p=0.425). However, a significant association (p=0.012) was observed if a participant’s father was a smoker; ESM was seen in 42.9% of these participants. A significant association (p=0.004) was observed for participants whose mothers were smokers; ESM was seen in 28.6% of these participants. A significant association (p<0.001) was observed for participants who had a sibling who smoked; of those who reported having a sibling who smoked, 10.7% had smoked a midwakh before. A significant association (p<0.001) was observed for participants who had a friend who smoked a midwakh; 54.8% of participants who had a friend who smoked a midwakh had also smoked a midwakh. Smoking any form of tobacco (other than with a midwakh) was significantly associated with midwakh smoking [χ^2^(1)=37.31; n=316; p<0.001]. The majority of students who smoked a midwakh (21.2%) had also smoked other forms of tobacco.

A multivariate logistic regression analysis was conducted to determine the factors associated with ever smoking midwakh, as seen in Table 2. Having a midwakh smoking friend was independently associated with odds of ever smoking midwakh that were 43 times more in those who had midwakh smoker friends than in those who had non-smokers friends (OR=43.27; 95% CI: 10.74–174.3; p<0.001). Having a friend consuming any kind of tobacco product other than midwakh was independently associated with ever smoking midwakh (OR=8.39; 95% CI: 1.15–61.36; p=0.036). Smoking any form of tobacco other than midwakh was also associated with an increased risk of ever having smoked a midwakh (OR=8.73; 95% CI: 2.05–37.16; p=0.003).

**Table 2:** Multivariate logistic regression analysis of factors associated with ever smoking midwakh

*Variable*	*OR*	*95% CI*	*p*
Age	0.30	0.08–1.19	0.087
Having a midwakh smoking friend	43.27	10.74–174.3	<0.001*
Having sibling who consumes tobacco	0.92	0.26–3.24	0.899
Having father who consumes tobacco	0.89	0.26–3.25	0.894
Having mother who consumes tobacco	3.31	0.58–19.05	0.180
Have smoking friend	8.39	1.15–61.36	0.036*
Consuming other forms of tobacco other than midwakh	8.73	2.05–37.15	0.003*

* Significant at p≤0.05. OR: odds ratio. CI: confidence interval.

## DISCUSSION

In this study, we estimated the prevalence of midwakh smoking and examined the role of sociodemographic variables in predicting midwakh usage among male college students. The percentage of participants in our study who had ever smoked a midwakh was 7.9%, and the percentage of those who currently smoked a midwakh was 3.8%. Additionally, Qassim is culturally a conservative region located at the center of the Kingdom of Saudi Arabia with no sea port and less tourism and intercultural mingling. These factors may lead to low prevalence and less reporting because of social desirability bias. However, the reported prevalence in the studies conducted in the UAE ranged from 12.5% to 39%^21-28^. The differences in the reported prevalence between these studies in the UAE may be related to sampling methods, location, and the genders and ages of the participants. It is clear that most of these studies conducted in the UAE showed a higher prevalence of midwakh smoking than our study. This could be explained by the popularity of midwakh in the UAE^26^. The geographical location of the UAE by the sea may also mean a stronger connection through trade with other countries, especially Iran where midwakh originated. A recent study conducted in Lebanon reported a midwakh smoking prevalence of 4.6%, which is higher than the percentage of current smokers in this study^29^. Despite the fact that the participants were younger than in our sample, the reported prevalence is higher than in our study. Furthermore, liberal culture, availability of midwakh’s products and tourism in the UAE and Lebanon could be reasons for midwakh popularity.

Regarding socioeconomic characteristics, we did not find any significant association between age, marital status, academic performance and income with ever smoking of midwakhs. This study only included students from a very narrow age range. Thus, there is little chance to see the effects of age on smoking. It has been found that as individuals grow older, they are less likely to be involved in risky behaviors such as drinking alcohol, smoking, and driving without a seatbelt^32^. Siddiqua et al.^28^ and Afifi et al.^29^ did not find any significant association between age and smoking midwakhs, and another study conducted among college-aged Saudi cigarette smokers and US waterpipe smokers did not find any association between age and cigarette smoking^8,33^. Regarding marital status and income, our findings are similar to a study conducted on predictors of smoking among Saudi university students^8^. In contrast, a study conducted in China found that married participants tend to smoke more cigarettes^10^. However, this study included a larger sample distribution, including many married participants, which is different from our student-focused sample. In terms of academic studies, the present study did not find any association between academic performance and smoking midwakhs, but a study conducted by Almogbel et al.^8^ found an association between academic performance and smoking cigarettes.

In the final logistical regression, we found that having a friend who smoked cigarettes or midwakh were associated with ever smoking midwakhs. Siddiqua et al.^28^ found an association between exposure to midwakh and smoking midwakhs. These results were consistent with other studies on cigarette smoking. One such study conducted on Saudi university students found that having a smoking friend was a significant predictor of being a smoker^8^. Similar results were found in studies conducted on adults in Jordan, China, and Taiwan who smoke both cigarettes and waterpipes^10,33,34^. This shows the effects of peer influence on initiating different types of smoking, including midwakhs^5^. This is an important finding as peers represent a level of influence for behaviors and can be used to promote healthy behaviors. Consuming tobacco in ways other than a midwakh was a significant predictor of smoking midwakhs. Other types of smoking were investigated in a study conducted by Afifi et al.^29^, who found a significant association between current smoking and smoking midwakhs. Two other studies also found that smoking other forms of tobacco was a significant predictor for cigarette or waterpipe smoking^10,35^.

### Limitations

One limitation of this study is that the results cannot be generalized, as the sample included only male students from one university. Additionally, all measures were self-reported, which may have introduced some biases such as social desirability bias. However, this may affect our results little as we did not collect any personal identifier of the participants. The cross-sectional design of the study also limits the ability to draw conclusions about the causality of midwakh usage.

## CONCLUSIONS

This is the first study that we are aware of that examines the sociodemographic determinants of midwakh use among male Saudi college students. Having midwakh smoking friends, having friends smoking any other form of tobacco, and being a smoker could predict the use of midwakh among university male students in Saudi Arabia. Interventions are required to control this behavior to prevent smoking-related health consequences. Further studies should be conducted to explore this form of smoking in different population groups including females and other age groups and any other predictors of midwakh use. The results of this study could help health educators and policy makers in planning appropriate measures to prevent this form of smoking getting a foothold among students in Saudi Arabia.

## CONFLICTS OF INTEREST

The authors have completed and submitted the ICMJE Form for Disclosure of Potential Conflicts of Interest and none was reported.
